# Impacts of an Intervention to Improve the Identification, Referral and Safety of Those Experiencing Domestic Violence: A Mixed Methods Study in the UK

**DOI:** 10.3390/ijerph192316181

**Published:** 2022-12-03

**Authors:** Shazia Zafar, Caroline Bradbury-Jones, Siddhartha Bandyopadhyay

**Affiliations:** 1 College of Social Sciences, University of Birmingham, Birmingham B15 2TT, UK; 2 School of Nursing, University of Birmingham, Birmingham B15 2TT, UK; 3 Birmingham Business School, University of Birmingham, Birmingham B15 2TT, UK

**Keywords:** domestic abuse, domestic violence, identification, interpersonal violence, mental health, mixed methods, referral, safety, deprivation

## Abstract

This study is the first evaluation of the impacts on long-term health issues (and associations with ethnicity and poverty) of a domestic violence intervention, Identification and Referral to Improve Safety (IRIS). IRIS is a domestic violence training, support and referral programme based mainly in primary care settings. This was a convergent, parallel, mixed methods UK study. In the quantitative phase, we matched the health records of 294 patients who had a marker for domestic violence with records from a domestic violence support service to track the health conditions of participants before and after referral to IRIS. In the qualitative phase, we conducted semi-structured telephone interviews with 21 women who had received IRIS support and thematically analysed the data. Descriptive statistics indicated that, at the point of referral to IRIS, participants had a variety of health conditions, with a reduction on a number of mental and overall physical health conditions post-IRIS. Qualitative data are reported under five prominent themes: life before, driving forces for help-seeking, experiences of support, perceived impacts and recovery as a journey. Overall, we found that IRIS support was associated with a positive impact on participants. The study highlights the benefits of improved identification and referral of domestic violence survivors.

## 1. Introduction

Domestic violence (DV) takes many forms and is characterised as controlling, coercive, threatening behaviour by those who are (or have been) intimate partners or family members, regardless of gender or sexuality. It is a significant global public health issue that is associated with substantial morbidity and mortality, reported to affect up to one in three women during their lifetime [1,2]. The World Health Organization [3] estimates that, globally, 27% of women aged 15–49 years have experienced physical and/or sexual intimate partner violence (IPV) at least once in their lifetime. In their report on abuse and violence against older women, the United Nations have highlighted the global significance of the problem [4]. In the UK, the year ending March 2020, the Crime Survey for England and Wales determined that women were significantly more likely than men to experience DV, with a respective prevalence rate of 8.1% and 4.0% [5]. The same survey reported that, in 74% of DV-related crimes, the victim was female, making DV a huge problem in the UK for both men and women.

Severe emotional distress, levels of depression, anxiety and post-traumatic stress disorder (PTSD) are well-known to be elevated amongst survivors of DV and IPV [6,7,8]. Moreover, for those who experience DV, the erosion of self-esteem through gaslighting and detachment from support networks causes reduced self-efficacy/confidence and loneliness [9]. Survivors of DV/IPV go on to suffer a myriad of health conditions, such as chronic pain, fatigue and gynaecological conditions impacting reproductive health, making specialised health services necessary [10]. As such, those who experience DV (mainly women), are most likely to approach and come into contact with healthcare services such as General Practitioner (GP, i.e., doctor) surgeries as the first point of contact, and in the UK, this is primary care.

Multiagency partnership working at an operational and strategic level is the most effective approach to addressing DV [11]. Within that partnership, primary care services are ideally positioned to play an important role in identifying, supporting and managing abusive experience [12]. Despite GP practices being well placed to support survivors and ameliorate the potential health consequences of exposure to DV, the recorded prevalence of DV exposure in primary care is substantially lower than expected [13], and barriers still exist for survivors wishing to report their experiences [14].

The identification and referral to improve safety (IRIS) programme is a well-established and evidence based DV training, support and referral programme for GP practices (medical surgeries) in the UK [15,16]. Due to the ever-changing nature of an intervention programme, information about IRIS can be found here: https://irisi.org/about-the-iris-programme/ (accessed on 18 November 2021). IRIS intervention includes practice-based training sessions, a prompt in medical records and a clear referral pathway to a named DV advocate educator (AE). The AE is an employee of Women’s Aid who receives specialised IRIS training to recognise and deliver the specialised support and needs of survivors of DV. The support can be provided at GP practices, Women’s Aid or the victim’s home. The AE also delivers training to GPs and primary care staff and ongoing communication/consultancy with them. Once trained, the GP practice identifies and refers victims of DV/IPV to IRIS, where the AE receives the victim. In the original study regarding the outcomes of IRIS, women attending intervention practices were six times more likely to be referred to an advocate; three times more likely to have a recorded identification of DV in their medical record; and 22 times more likely to have a conversation about DV with their health care professional than those attending the control practices [15]. Since the original study (which was based on 24 women and 24 controls), a number of local evaluations of IRIS have taken place [17,18]. However, the evidence around the impacts of the intervention for DV survivors in terms of their health or differential impacts related to markers of inequalities, such has deprivation, have been less well explored. This is the first time the IRIS programme supporting those suffering from DV is being evaluated for its impact on health measured with deprivation. “Deprivation” is the term used to describe poverty in areas of England and data pertaining to it is collected and recorded officially every 10 years by the UK government. The data provide levels of poverty and affluence recorded as deprivation, either least deprivation or most deprivation (grouped in deciles), of areas of England that are based on access to education, employment, healthcare and housing, amongst some of the parameters. It is an important aspect to measure because the more deprived areas of England do not have the same access to healthcare as the more affluent areas. The lack of access would mean that those suffering from DV would not be able to access support services specifically designed to provide the multiagency support in primary care. As such, it is vital to understand how the IRIS service is being utilised in primary care and by whom.

The aim of this study was to investigate the impact that IRIS had on health outcomes (focusing on longer-term outcomes); how survivors perceived this impact; and the relationship between IRIS support, its outcomes and deprivation.

The specific research questions were as follows:

1. What impacts does IRIS have on recorded health conditions as captured in GP and IRIS data?
2. How do survivors of DV who have accessed IRIS support describe its impacts on their health and well-being?
3. What is the relationship between IRIS support, its outcomes, who survivors are and deprivation profile?
4. How do the quantitative and qualitative strands of the study interact to inform future research and practice?

This study provides important information about victim’s health both before and after the intervention of support to survivors of DV/IPV, and this is the first time the data are being mapped alongside information on deprivation in England. This will help to form a greater understanding of how effective the intervention has been, which ethnicities have accessed it and whether it has reached those who live in more or less deprived areas of England.

## 2. Methods and Materials

**Figure 1:**
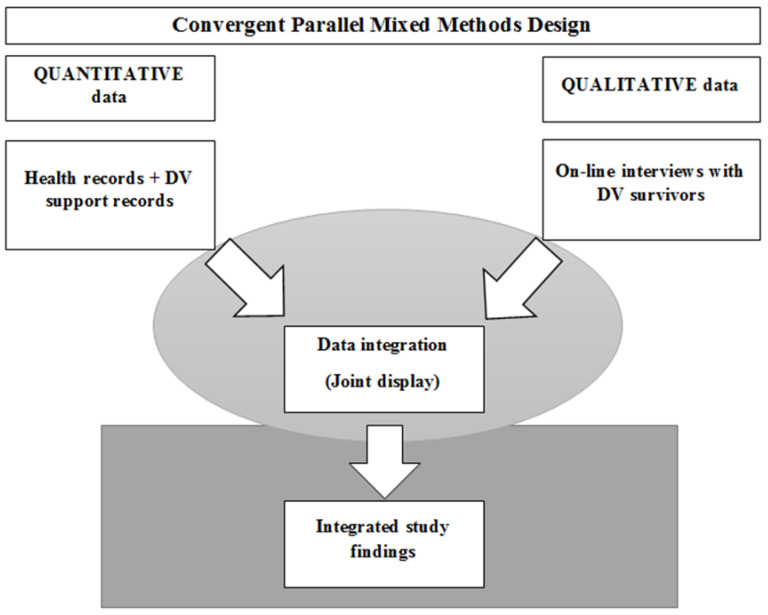
Schema for the mixed-methods research design.

The study took place between March 2019 and February 2021 in one large urban conurbation (West Midlands) in Central England, UK. Birmingham, Sandwell, Dudley, Walsall, Solihull and Coventry were included in West Midlands conurbation. The study was mixed methods, with a convergent, parallel design [19]. The intention in this type of study design is to use different but complementary data that, when triangulated, provide a deeper understanding of the phenomenon of interest than would be achieved with one approach alone. Data from both phases were analysed separately and then integrated by using joint displays of quantitative outcomes presented against themes from the qualitative interviews (see integration section of this article). The schema for the study is presented in Figure 1.

In the quantitative phase, GP records of 294 patients were matched with records from AEs to obtain a picture of the types of health conditions the women suffered from, including information on lifestyle, pregnancies, abuse and violence currently suffered and historical abuse, as well as the support IRIS provided. Their demographic information was collected, and postcodes (Zip codes) were used to extract a deprivation score of each patient whose records were accessed for the purpose of data extraction. The health conditions were recorded before and after IRIS intervention. A pre- and post- analysis was then conducted regarding the presence or absence of health conditions before and after IRIS support. The qualitative phase comprised semi-structured interviews with 21 women who had received IRIS support and consented to participate in the study. Full ethical approval was granted by University of Birmingham Science, Technology, Engineering and Mathematics Ethical Review Committee (ERN_18-1242).

### 2.1. Quantitative Phase

#### 2.1.1. Data Collection

All GP practices in the West Midlands who had received IRIS training were identified as sites for inclusion in the study and were sent a copy of the ethics letter, as well as a study documentation to participate in the evaluation study. Customised spreadsheets for each practice containing anonymisation codes (per patient) were also sent, alongside details explaining how to collect the data.

The study team collected data from GP practices and IRIS support agencies (Women’s Aid) across the West Midlands region. Data for all referred patients who were included in this study were extracted from patient health and support records according to variables pertaining to socio-demographic (including age, children in household, ethnicity and postcodes), lifestyle, prescriptions, consultations, pregnancies, health conditions, mental health, abuse and violence suffered and historical abuse, as well as the referral to IRIS and support and the nature of support provided.

A pre- and post- analysis was undertaken on data from GP practices pertaining to recorded health conditions to discern any statistically significant changes. This information was used to map any changes in recorded health conditions once IRIS support had been delivered. Pre-data: Data were collected from the timepoint 12 months prior to the IRIS referral date. Post-data: Data were collected from the IRIS referral date until the date of data collection.

Inclusion criteria: IRIS-trained GP practices where referral of patients to IRIS had taken place; patients aged ≥ 18 years; and all referrals to IRIS, including self-referrals and re-referrals within the inclusion time points (as above). Exclusion criteria: If IRIS support had not taken place (i.e., if a patient had declined support or left the GP practice or other reasons which rendered IRIS support not provided); and patient referred to IRIS from 1 January 2020 onward. Prior to data collection two pilot studies were conducted to understand the GP health systems database and the logistics of working collaboratively with GP practices and IRIS support agencies. The pilot findings helped to test the operational aspects, including the sampling procedures and processes for data retrieval.

#### 2.1.2. Sampling Procedure

To ensure anonymisation, an 8-step process was developed:

1. The IRIS support agencies sent the researcher team a list of referrals from each GP practice and the training dates of GP practices from each area.
2. The research team identified GP practices for inclusion in the study and provided the IRIS support agencies with a list of GP practices and numbers of referrals from each.
3. The agencies added the anonymisation codes and referral dates of each patient from each of the GP practices and sent to the research team.
4. Agencies sent names of the patients to GP practices. In doing so, this kept the names of the patients blind to the research team.
5. The research team used agency anonymisation codes for each patient and created customised blank spreadsheets for each patient and sent to each GP practice for data collection across the study area.
6. The research team created a customised blank spreadsheet for each patient/referral for data collection on the support provided and sent to all IRIS support agencies.
7. GPs returned to the research team the collected data by return of spreadsheets (from Step 5) on health information for each patient/referral.
8. Agencies sent the research team the data on IRIS support provided to each patient/referral by return of spreadsheets (in Step 6).

This 8-step process allowed agencies to work with the research team in an anonymised way, thus adhering to our rigorous procedures to protect identity.

#### 2.1.3. Data Analysis

An analysis was undertaken on demographics for descriptive statistics, including the ethnicity of patients, and then mapped with deprivation. This allowed for an investigation of the differences between the participants and whether there was variation in the participants who were offered IRIS support, so a pre- and post- method was used. Health conditions were recorded (with research team aiding the healthcare staff in data collection) before and after IRIS intervention, with an analysis conducted on aggregated data. A pre- and post- analysis of health conditions was undertaken as a crude measure of whether patients had less health complaints post-IRIS. A statistical analysis, including *t*-tests for statistical significance, was conducted on the data set, using Excel. A deprivation analysis was conducted by using data from the tool 2019 Index of Multiple Deprivation (IMD) [20] and was mapped onto health data.

### 2.2. Qualitative Phase

#### 2.2.1. Recruitment

All women were identified through an AE who was familiar with the woman and who was able to assess her safety to participate. All women who were identified as potential participants were provided with a Letter of Invitation and an Information Leaflet. The study information included details of participants’ ability to withdraw from the interview at any point or to remove their interviews from the study up to two weeks following interview (no women opted for either of these actions).

#### 2.2.2. Data Collection

Qualitative telephone interviews were conducted with 21 women who met the inclusion criteria. The interviews were conducted by two female members of the research team SZ, CB-J following blind peer review) with reference to a preprepared interview schedule and topic guide, which was used reflexively according to the nature of the interaction between interviewer and participant. The topic guide with included broad questions to stimulate participants to discuss the support they had received and how that impacted their lives before and after IRIS support. Data collection took place between June and September 2020. Oral consent was recorded at the beginning of each interview. All interviews were audio-recorded with the participant’s consent. Duration of interviews ranged from 10 to 58 min. Twenty interviews were undertaken in English and one in Urdu. The 20 interviews conducted in English were transcribed by a professional transcription service. The one Urdu recording was translated and transcribed into English by the English/Urdu-speaking research team member. To protect anonymity, each participant was assigned a code comprising a letter (donating the area of residence within the region) and a numerical identifier.

#### 2.2.3. Data Analysis

The transcribed interviews were analysed by using the thematic approach described by Braun et al. [21]. NVivo was used to thematically analyse the transcripts grouping into themes. This is a well-utilised approach in qualitative analysis because of its stepwise and robust approach. The approach is dependent on deep emersion in the data, thus aiding familiarisation. To achieve this, two researchers read and re-read the transcripts, independently, in a blind peer-review. One researcher then undertook the initial analysis and subsequently met with the second researcher to discuss the rudimentary themes. These were discussed and revised until both researchers agreed the themes as presented in this paper.

## 3. Results

### 3.1. Quantitative Results

A total of 156 GP practices were identified for potential inclusion in the study. From these, 47 responded with data collected from 294 patients. There were 30 males included in this sample. The age range was 18–91 years, with a mean age of 42 years. There were 22% who were 18–29 years, 31% were 30–39 years, 22% were 40–49 years and 13% were 50–59 years. Those over 60 (60–89 years) formed 13% of the sample cohort, along with one participant who was 91 years of age. The study has shown a wide range of ages, and, in particular, those over 60 years of age were lower in numbers in comparison to those of younger years (see Table 1). Although further socio-demographic information was requested, such as marital status, education, employment and household members, these data were not available; these data were not recorded on the patients’ medical records and were not always recorded by the support agencies, unless such data were pertinent to the support provided. Table 1 shows the ethnic backgrounds of all 294 patients, with the majority (55%) being of White British background, and the next highest in percentage was British Pakistani (11%).

#### 3.1.1. Mapping Deprivation

**Figure 2:**
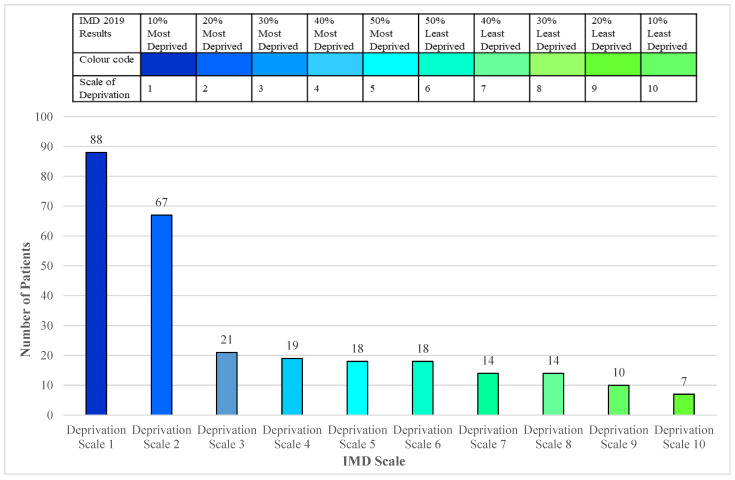
Deprivation profile of included patients (*n* = 276).

The IMD was used to establish relative deprivation across the study area, as this is the official measure of relative deprivation for small areas in England. Using the IMD tool [20], a scale of deprivation was created, where deciles of 10% to 50% of the most deprived areas of England were classed as 1–5 for ease, and deciles of 50% to 10% of least deprived areas of England were classed as 6–10 for ease, as seen in Figure 2.

As shown, the number of patients (32%) who fall in 10% of the most deprived decile. This decile forms England’s most deprived areas. The next highest percentage of patients (24%) fall in the 20% of the most deprived decile. The results indicate that collectively, 77% of patients in the study area lived in the most deprived areas, which is represented by decile scales of 1 to 5. The rest (23%) lived in the least deprived areas of England represented by decile scales of 6 to 10.

**Figure 3:**
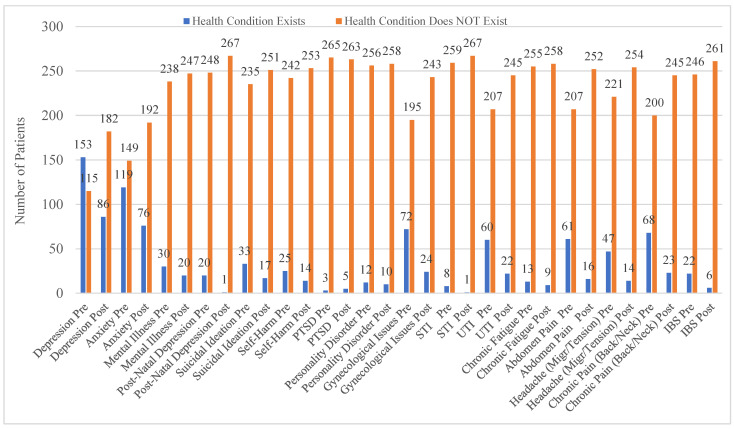
Recorded health conditions pre- and post-IRIS (*n* = 268). (PTSD = post-traumatic stress disorder, UTI = urinary tract infection, STI = sexually transmitted infection, IBS = irritable bowel syndrome, Migr = migraine).

Figure 3 shows the presence of health conditions before and after IRIS support as reported in GP records of 268 patients (26 patients excluded from this part of the analysis due to incomplete health data). This overall profile of aggregated results indicates that there was a difference in all health conditions before and after receiving IRIS support. Depression, anxiety, mental illness, post-natal depression, suicidal ideation and self-harm all show decline after IRIS intervention. As indicated in the results, pre-IRIS, 57% (*n* = 153) of the sample had depression recorded and 44% (*n* = 119) had anxiety recorded. Post-IRIS, depression and anxiety were recorded at 32% (*n* = 86) and 28% (*n* = 76), respectively, indicating decline.

For the 268 patients included on the health conditions analysis, further analyses were undertaken to map deprivation on those patients for whom deprivation data were available (250 out of the total of 268) in relation to their specific health conditions. From a total of 294 patients in the overall sample, missing data for different parts of the analysis were as follows: missing postcodes in the deprivation mapping meant that 276 patients were included (with 250 included in the health profiling); pre- and post-analysis included 268 patients due to missing data.

#### 3.1.2. Health Conditions and Deprivation Analysis

**Figure 4:**
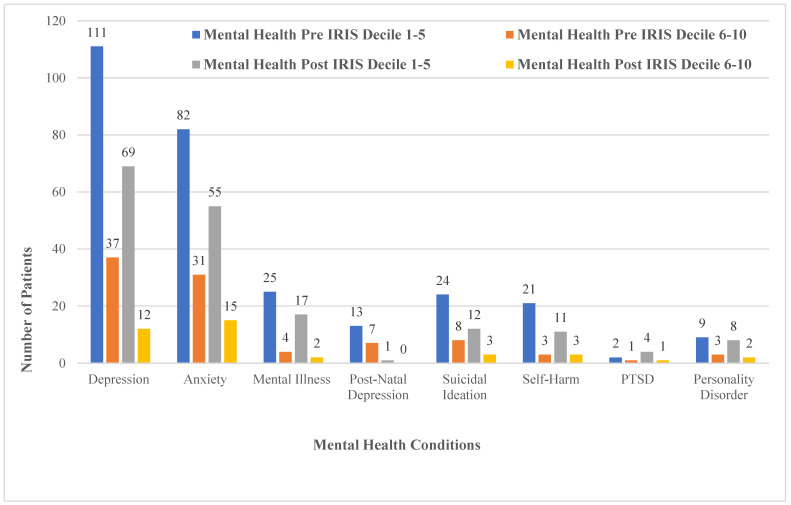
Comparison of mental health conditions and deprivation decile (*n* = 250).

Figure 4 compares recorded mental health conditions and deprivation decile. Pre-IRIS in decile 1–5 (the most deprived areas), 44% (*n* = 111) of the sample had depression recorded, and 33% (*n* = 82) had anxiety recorded. Post-IRIS in deciles 1–5, depression and anxiety which were recorded at 28% (*n* = 69) and 22% (*n* = 55), respectively. Figure 4 also illustrates the health conditions mapped with deciles 6–10, the least deprived areas. Pre-IRIS, 15% (*n* = 37) of the sample had depression recorded and 12% (*n* = 31) had anxiety recorded. Post-IRIS in deciles 6–10, depression and anxiety were recorded at 5% (*n* = 12) and 6% (*n* = 15), respectively. Suicidal ideation was recorded at 13% pre-IRIS, with a drop to 6% post-IRIS, and when mapped for decile 1–5, pre-IRIS was recorded at 10%, dropping to 5% post-IRIS. Similarly, in deciles 6–10, 3% recorded pre-IRIS and 1% recorded post-IRIS.

**Figure 5:**
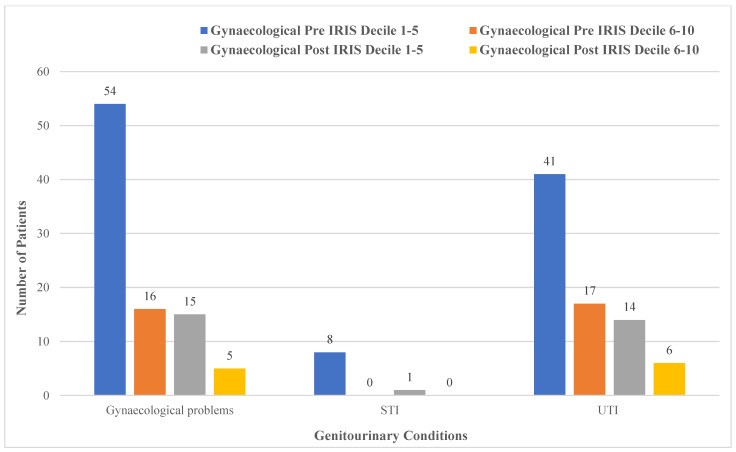
Comparison of genitourinary conditions and deprivation decile (*n* = 250).

Figure 5 shows the genitourinary health conditions of the 250 patients related to deprivation decile. The health data were mapped according to decile information, and all indicated a decrease post-IRIS intervention. Pre-IRIS in deciles 1–5, 22% (*n* = 54) of the sample had recorded the broader gynaecological conditions, 3% (*n* = 8) was recorded for STIs and 16% (*n* = 41) recorded for UTIs. Pre-IRIS in deciles 6–10, 6% (*n* = 16) had gynaecological conditions, 0% had STIs and 7% had UTIs recorded. Post-IRIS in deciles 1–5 gynaecological conditions, STIs and UTIs were recorded at 6% (*n* = 15), 0.4% (*n* = 1) and 6% (*n* = 14), respectively. Post-IRIS 6–10, gynaecological conditions, STIs and UTIs were recorded at 2% (*n* = 5), 0% and 2% (*n* = 6), respectively.

**Figure 6:**
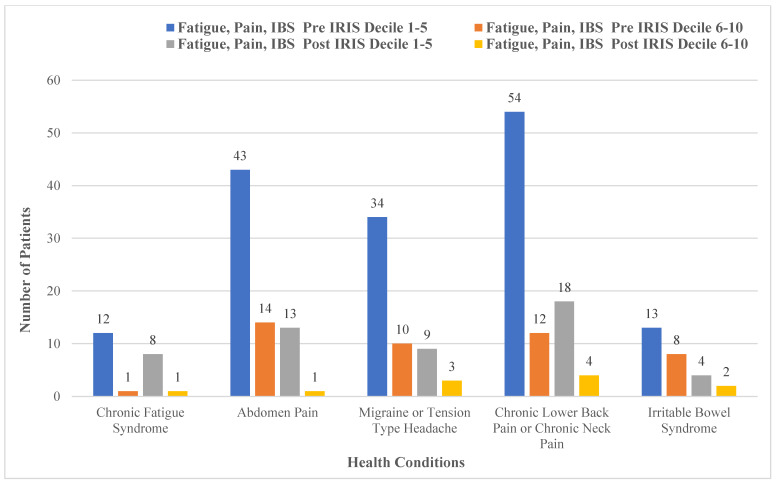
Comparison of fatigue, pain and irritable bowel syndrome and deprivation decile (*n* = 250).

Figure 6 illustrates the health conditions associated with pain, fatigue and irritable bowel syndrome (IBS). The health data were mapped according to decile information of the 250 patients, and all dropped post-IRIS intervention. Although Figure 6 outlines specific health conditions associated with pain, these conditions are portrayed collectively as pain in the following recorded data. Pre-IRIS in deciles 1–5, 52% (*n* = 131) of the sample had recorded pain, 5% (*n* = 12) for recorded for chronic fatigue and 5% (*n* = 13) recorded for IBS. Pre-IRIS in deciles 6–10, 14% (*n* = 36) had pain recorded, 0.4% (*n* = 1) chronic fatigue and 3% (*n* = 8) had IBS recorded. Post-IRIS in deciles 1–5, pain, chronic fatigue and IBS were recorded at 16% (*n* = 40), 3% (*n* = 4) and 2% (*n* = 4), respectively. Post-IRIS in deciles 6–10, pain, chronic fatigue and IBS were recorded at 3% (*n* = 8), 0.4% (*n* = 1) and 1% (*n* = 2), respectively.

In summary, the average number of health conditions per patient included in the study was 2.78 pre-IRIS, and post-IRIS, the average number of conditions per patient was 1.28. The statistical analysis included two-tailed *t*-tests, with a cut-off for significance of 0.05 used to reject the null hypothesis and the *p*-value obtained lower than 0.05. A Mann–Whitney U-test was also conducted where the U stat value is 73 and lower than the U critical value of 83, so the difference is significant, with both statistical tests confirming significance. This is based on aggregated results for the overall study area. The results indicate an overall improvement of mental health conditions collectively, as well as in those conditions associated with pain. Depression pre-IRIS was recorded at 59%, and post-IRIS, it was 33%; anxiety pre-IRIS was recorded at 44%, and post-IRIS, it was 28%, suicidal ideation pre-IRIS was recoded at 13%, and post-IRIS, it was 6%; and overall pain was recorded pre-IRIS at 66%, and post-IRIS, it was 19%.

Both pre- and post-IRIS, the analyses highlighted that a higher number of patients suffering with the health conditions were living in the most deprived areas (deciles 1–5) of the West Midlands, England. In comparison, those living in the least deprived areas (deciles 6–10) who suffered the health conditions were lower in numbers. Mental health conditions overall were recorded to have a mean of 47% in deciles 1–5 pre-IRIS and 30% post-IRIS, whilst in deciles 6–10 pre-IRIS, the average of overall mental health conditions was 16% pre-IRIS and post-IRIS at 7%. The average percentage of overall gynaecological problems were 65% in deciles 1–5 pre-IRIS and 14% post-IRIS, whilst in deciles 6–10, the overall average was 12% pre-IRIS and 4% post-IRIS. The symptoms of fatigue, pain and IBS collectively showed an overall average of 57% in deciles 1–5 pre-IRIS and 21% post-IRIS, whilst in deciles 6–10, the overall average was 17% pre-IRIS and 5% post-IRIS.

The results provide a promising picture of overall improvement in health conditions when IRIS intervention is provided. However, the results need to be interpreted with caution because they are based on aggregated data from a single cohort of participants in one geographical region of England. Our results are also presented with the caveat that the absence of a health condition recorded at a point does not mean the absence of the condition. This is particularly true for chronic conditions.

### 3.2. Qualitative Findings

Sample Characteristics: The women’s ages ranged from 22 to 58 years, with a mean age of 40.6 years. The sample reflected diversity as regards ethnicity and religion. Women self-reported as mostly heterosexual (with two identifying as lesbian). Two women self-reported as disabled. The findings are presented under five themes: life before, driving forces for help-seeking, experiences of support, perceived impacts and recovery as a journey.

#### 3.2.1. Life Before

To obtain a sense of the impacts of IRIS, a qualitatively derived baseline was established by asking participants about their health and well-being prior to accessing IRIS support. All participants gave an account of significant psychosocial challenges, as shown in the following examples. These reflect the serious impacts of controlling behaviours, isolation and ‘gas lighting’:

*I almost lost emotion, but I also lost the ability to be able to control emotion… I’d feel more anger than I’ve ever noticed I’ve ever felt before… I struggled quite a lot with that and there was a lot of emotions that I was unable to control, almost.*

(W5)

Participant D2 echoed such experiences of being ‘ground down’, also highlighting the additional strains of being a parent:

*Yeah, I just felt really low like and, to be honest, I think I was just ground down by it all. You know, police intervention, child services, I mean I was a bit embarrassed. You know the last thing you want as a parent is child services—you feel like you’ve failed a little bit, do you know what I mean?*

(D2)

#### 3.2.2. Driving Forces for Help-Seeking

As part of the interviews, we were keen to learn about prompts to help-seeking and the factors that led women to access IRIS support. In the context of their own experiences, many participants explained their reasons:

*Things got really bad for me and I needed some support and didn’t understand a lot of things. Some things had happened which was really hard for me to understand and I needed the right medication and I needed the right support and help and I think that’s when [AE] kind of came in a supported me in my hospital appointments and anything else that I needed really.*

(W8)

One participant recalled the supportive actions of her GP that had helped her to realise the abusive nature of her relationship (that subsequently led to a referral to IRIS):

*I told the doctor, and I just explained the situation, how it was at home, because how I was living with him. And it was the doctor who diagnosed it as coercive control.*

(D7)

One participant explained that the absence of potential barriers was instrumental in her help-seeking:

*Once I knew that it was only down the road, for example—there was lots of things that helped me make that decision, so the fact that it was local, that I could do it, it was term time only, they had child care… so in a way… it got to the point that I couldn’t not go! Because there were no barriers, you know? So, all the barriers that, you know, would stop me from going were sorted out.*

(B4)

#### 3.2.3. Experiences of Support

Given the focus of the study, it was inevitable that a great deal would be heard from the participants about the IRIS support they had received. We present here some of the salient points and insights gained. Most participants talked of the specific support offered by their AE:

*She’s just sat there and allowed me to speak, allowed me to cry, allowed me to express. And she’s given me all… support and advice.*

(D6)

*She was very good with just speaking to me about gaining control because I felt that I’d lost all control… She gave me all the right help at that time so I think it was a really good outcome… I don’t know which place I would be in if I hadn’t met [AE]. I probably would have been in a lot of a worst place.*

(B5)

In the excerpt from participant B5 above, the issue of gaining control is discussed. This resonated with the accounts of so many women. IRIS support allowed women to understand the nature of their abuse from a more objective viewpoint, which is important in mitigating the effects of ‘gas lighting’:

*Yeah, it was really good, because I think when you’re in a domestic violence situation and you’re clouded by a lot of factors, it’s kind of, you know, someone shedding that bit of light on it for you so you kind of look at it in a different way, if that makes sense?*

(D2)

Many participants talked of the importance of being listened to and, as illustrated in the quotes below, the essential act of validation:

*And I think it’s very important that you are validated by professionals. Not that other women can’t validate you, that’s also important. But I think that it’s really, really important that you can get to an understanding yourself that what was done to you was wrong, and that you’re in no way responsible for it.*

(B4)

*And I think, I just needed that validation I think, and to believe myself. Because there was lot of things that I didn’t say out loud, there was a lot of things that I didn’t act on, because if I said it out loud it was real. I think saying it out loud and realising it’s real is one of the most important things because otherwise it just gets hidden away and ignored.*

(W5)

We have already reported on the importance of accessibility. Our sample was predominantly made up of women participants for whom English was their first language. Crucially though, we learned of the support available to non-English speaking participants that facilitated their access and utilisation of support:

*She [AE] told me, ‘don’t worry if you can’t speak English, just call me and if you can’t speak, just ring and say “call me”’. She said she would call back with an interpreter. I would message her ‘call me’ and she would call back with an interpreter who could speak Urdu… then without fail I would receive a call from her. So that’s why I say that even my soul prays for her. I received a lot of help from [AE].*

(B1)

#### 3.2.4. Perceived Impacts

In this section, we highlight the impacts of IRIS support of participant’s perceptions of health, particularly their psychological and emotional well-being. First, we present a quote that shows the impacts of IRIS on shaping women’s understandings, not only of the abuse itself, but on awareness of psychological help as opposed to the prescription of antidepressants and what happens post-referral (which is often a barrier to help-seeking):

*I think to be honest with you in that aspect, it’s… everyone’s aware of antidepressants. Nobody is aware of anti-abuse workers. Forget the antidepressants, no one is aware that we have support workers out there who can do a lot better than those antidepressants can.*

(D6)

The positive impacts of IRIS were evident in all participants’ accounts, and participants expressed this in multiple ways. For some participants, the impacts related to physical health, mobility and improvements in self-reported eating disorders, as illustrated in the following accounts:

*My diabetes, well, that is much, much better and my physical health, I’m getting fitter, I’m walking more, I’m exercising more. I’m taking more care of me. That’s the thing, I never took care of me and now I am. So, it’s had a massive, massive impact on every aspect of my health.*

(D1)

*Well, I was in a wheelchair… and now, the wheelchair is there if I need it, but I’m—I’m trying to make myself…I push myself to go down for a walk to the garden.*

(W6)

**Figure 7:**
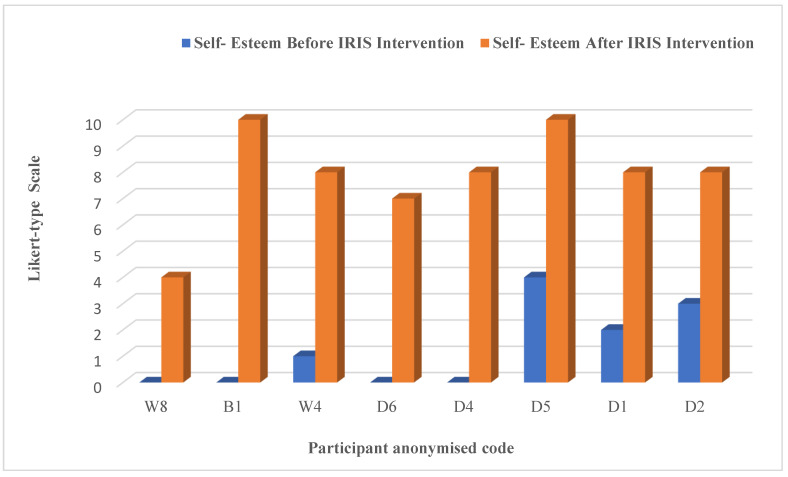
Self-reported esteem of women interviewed in the qualitative phase, using Likert-type scale.

In most of the interviews (where it seemed appropriate), the participants were asked to assess their sense of self-esteem before and after IRIS and score it based on a Likert-style scale, with 0 relating to extremely poor and 10 being extremely high. Figure 7 reflects some of their responses to this qualitative self-rating.

As shown in these eight reflections, the perceived impacts of IRIS were considerable. Perhaps unsurprisingly, freedom from control and gas lighting were reported by most women as being powerful impacts of IRIS:

*I started reading books because he wouldn’t let me read books… do you know what I mean?*

(W6)

*I feel, I’m beginning to feel freer than I ever felt, I’m not looking over my shoulder as much (although I am looking over my shoulder), but it’s definitely not the way I did it before when it first happened.*

(S4)

#### 3.2.5. Recovery as a Journey

In spite of the evident positive impacts, it is important to recognise that recovery from abuse is a process. It does not occur on a linear timeline, and it takes considerable time. Several participants captured this in their narratives, many using the word ‘journey’ to refer to their own recovery (such as B4):

*I haven’t come to the end of my journey in terms of dealing with what happened to me, but I’m a lot further forward than I would have been, had I not been able to get outside support. It’s taken a long time for me to recognise and understand that my mission in life is to be the best version of me.*

(B4)

As the following three accounts demonstrate, even on a trajectory of recovery, there are periods of ‘down days’ (D6), ‘worries’ (D2) and ‘difficult times’ (D1). This accounts for why sometimes receiving support means that women may need more help from their GP initially, rather than less, as part of the recovery process. The importance is in the trajectory toward healing:

*I mean, don’t get me wrong, I do still have my down days, and I’ve had moments where I’ve been on the antidepressants but then I put my children first and think, well if I’m teaching my children that they need to battle to succeed in their life, me as being their role model and being on antidepressants—what am I actually teaching them? I’m totally going against my own teachings.*

(D6)

*I think my outlook is completely different and can actually say that I’m happy. Don’t get me wrong, I have my worries and… about the future, you know, it is daunting being a single parent, you know, it is hard work but I do have good support network and my boys are really happy, you know, because I’m happy they’re happy you know?*

(D2)

To obtain a sense of a continued journey, where appropriate, we took the opportunity to ask participants to consider the future. The following excerpts are participants’ responses to the following question: Where do you see yourself in about a year’s time?

*I’ve enrolled into college to start in September... I’ve got my goals, in place, where I want to get to. You know I want to study, I want to further my career, I want a secure future for my children.*

(D2)

*I’ve got my life back on track and I’m feeling a lot better about myself.*

(D6)

Overall, the qualitative phase highlighted the very clear benefits on IRIS according to the subjective experiences of the women who took part. In the following section, we explore these findings in juxtaposition to the quantitative results.

### 3.3. Data Integration

The advantage of a mixed-methods study design is that it allows the inherent limitations of quantitative and qualitative approaches, as separate entities, to be mitigated, through evaluating both sets of data side by side. To capture these juxtaposed insights, we developed a data-integration table (Table 2).

**Figure 8:**
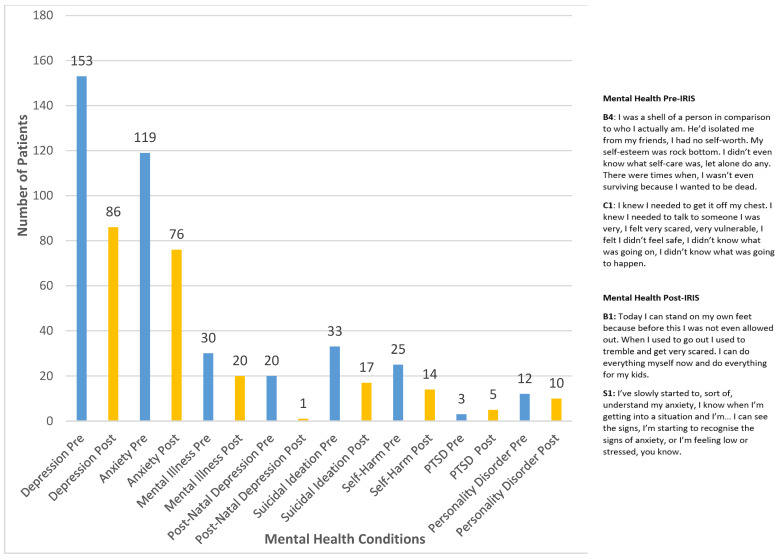
Mental health conditions existing pre- and post-IRIS intervention and mental health captured during qualitative phase. When taken together, the insights from both strands of this mixed-methods study point to a potential decline in overall health conditions after IRIS support is provided. Although there are limits to the inferences that can be drawn from a mixed methods perspective, it does not however detract from the importance of the quantitative results in isolation and opens opportunity for further qualitative and or mixed methods research in these areas. Therefore, we suggest a further study encompassing aspects from this paper targeting a larger participatory cohort. As regards deprivation, we gleaned important insights from the quantitative results, but we did not collect demographic data in the qualitative phase in terms of women’s socio-economic status. While this may have been of interest to show a relationship between the quantitative and qualitative strands, we do not regard it as a limitation to the usefulness of the study results.

As shown, the integrated findings show that the qualitative findings supported the quantitative results in relation to mental health conditions. Genitourinary conditions and fatigue, pain and IBS were not discussed explicitly by participants in the qualitative phase; they felt that there was a positive impact on their overall health. Specifically, the women from the qualitative phase pointed toward their affected mental health and spoke of how the IRIS intervention provided support which helped with their anxiety and depression. Figure 8 provides a juxtaposition of the quantitative results and qualitative findings, showing convergence of both phases in relation to impacts on mental health.

## 4. Discussion

Our combined results from this mixed-methods study showed that IRIS appeared to support some of the most deprived participants based on the sample we analysed, with 77% belonging to the most deprived deciles 1–5 (and 23% in the least areas of deprivation).

The quantitative phase of the study showed a drop (from 2.78 to 1.28) in the mean number of health conditions recorded. Depression, anxiety, mental illness, post-natal depression, suicidal ideation, self-harm, pain and genitourinary conditions all showed up with a lower frequency in the data following IRIS support, indicating an improvement in overall health and that IRIS has been therefore useful (albeit with the caveats already discussed). The wide reach in survivors from different ethnic backgrounds shows that IRIS had been used in providing support to those from varied backgrounds. Furthermore, the greatest number of survivors in this study lived in the most deprived areas and suffered with the highest levels of health conditions pre-IRIS (average mental health, 47%; gynaecological issues 65%, fatigue, pain and IBS, collectively 57%). Their health conditions indicated improvement post-IRIS (average mental health, 30%; gynaecological issues, 14%; fatigue, pain and IBS, collectively 21%). In comparison to the least deprived areas the numbers suffering health conditions were lower overall (average mental health, 16%; gynaecological issues, 12%; fatigue, pain and IBS, collectively 17%), and the study indicated improvement post-IRIS (average mental health, 7%; gynaecological issues, 4%; fatigue, pain and IBS, collectively 5%). However, we do note that the absence of a chronic condition on a GP record may not indicate that the condition is no longer present and, thus, the recorded drop in various forms of mental ill-health must be interpreted with caution. Further, given the absence of data on a control group, these relations should be looked at as interesting correlations rather than interpreted causally. However, with the added evidence from the qualitative phase, these correlations support the hypothesis that IRIS had a positive impact on the health of those who received support.

The quantitative data for mental health conditions when mapped to deprivation scale showed improvement in patients from the most deprived areas. These results support the findings from the qualitative phase, where women spoke of improvement in mental health conditions following IRIS support. Our quantitative results are consistent with rates recorded nationally, with 59.1% of women referred to IRIS experiencing mental ill health, mainly depression and/or anxiety [18]. The study findings regarding mental health impacts are important when considering the broader DV literature. The long-term impacts of DV on mental health are well established [6,22,23]. In a US-based longitudinal study, Zlotnick et al. [24], found that women who experienced DV continued to experience long-term mental health problems regardless of whether or not they remained in an abusive relationship. Furthermore, evidence suggests that the relationship between DV and psychological ill-health is bidirectional; that is, women who experience mental health difficulties are at greater risk of DV, and vice versa [25].

As regards other recorded health conditions reported in this paper, the quantitative results showed a drop in recorded symptoms of chronic fatigue syndrome, pain and irritable bowel syndrome. The results align with those of previous studies, where intimate partner violence and the risk of developing fibromyalgia and chronic fatigue syndrome have been reported [23]. While women in the qualitative phase did not talk extensively about pain, this was probably because they were not directed to do so within the interview guide. This provides opportunities for further qualitative exploration that focuses specifically on the association between DV and pain. Similarly, as regards DV and genitourinary problems, a well-reported relationship exists, for example, in relation to painful bladder symptoms [26] and sexually transmitted infections and HIV infection [27]. This association was confirmed in our study, but importantly, demonstrating a drop post-IRIS support. For a detailed observation in individual health of participants who receive IRIS support, we recommend further study.

Whilst our study illustrated the positive benefits to overall health in patients referred by GPs to the IRIS programme, this was very much dependant on GP surgeries having had IRIS training. Identification and referral responses that lead to support from AEs are contingent on health professionals being aware of the nuanced indicators of DV and who know the referral pathways. Up-skilling the healthcare workforce in relation to recognition and response to DV is crucial and is an issue that has gained prominence in the literature over recent years. Despite the fact that women experiencing DV are frequent users of primary care health services [28], health professionals’ responses to disclosures of DV are inconsistent. In a UK-based qualitative study, Keeling et al. [29] found that, while some women reported receiving appropriate support following a disclosure, others described experiencing dismissal and renunciation from health professionals. Similarly, Ramsay et al. [30] found that primary care clinicians in the UK felt ill-prepared to enquire about and respond to DV, with many reporting insufficient previous training as a contributing factor. All of these (studies) suggest the need for a specialised service. The findings highlight the importance of trained primary care practices that can provide timely referral to such services, who act as the first point of disclosure for referral and safety planning.

There were some limitations to the study, mainly in regard to the collection of data during the quantitative stage due to the national lockdown enforced due the COVID-19 pandemic. Although we were able to sample patients referred to IRIS across practices in the conurbation of the West Midlands, the response by medical surgeries was lower than anticipated. Hence, there is the possibility that a larger number of patients were referred to IRIS for support. The impact due to lockdown made it less possible to obtain data for a control group to compare to a treatment group. While we do not read too much into the post-data for chronic health conditions, with the caveat that for chronic conditions an absence would not necessarily indicate an absence of the condition, it is encouraging to find that even non-chronic conditions such as UTI and STI show a reduction post-IRIS. The problems with data capture suggest that some changes in accountability structures may be needed, with an expansion of services coming with a duty to participate in an evaluation, as under the present governance structure, GPs’ participation in evaluation studies and research is discretionary.

## 5. Conclusions

A pre- and post- analysis was conducted on the presence or absence of conditions before and after IRIS support, and the results indicated an improvement in overall health conditions. We found that women who engaged with IRIS during the study period had positive impacts on their health in the short term and that participants perceived that these would be long-lasting. Our findings support the current literature suggesting the positive outcomes of IRIS, and we recommend the continued delivery and upscaling of the programme, modified as required to meet shifting global impacts.

## Figures and Tables

**Table 1:** Profile of participants (*n* = 294) according to recorded ethnicity, age and gender.

Recorded Ethnicity	Participants (*n* = 294)
White British	163
British Pakistani	32
British Indian	15
British Asian	13
British Other	11
European	8
White Other	7
British Bangladeshi	6
British African	2
British Caribbean	2
White and Black African	3
White and Black Caribbean	1
White Irish	1
Other Mixed Background	1
Other	18
Blank	11
**Age (years)**	
18–29	57
30–39	80
40–49	59
50–59	34
60–69	17
70–79	16
80–89	2
90–100	1
**Gender**	
Female	264
Male	30

**Table 2:** Mixed-methods data-integration table.

Domain	Quantitative Results	Qualitative Findings	Inferences	Implications
**Mental health conditions**	Pre-IRIS, 57% (*n* = 153) of the sample had depression recorded, and 44% (*n* = 119) had anxiety recorded. Post-IRIS, depression and anxiety were recorded at 32% (*n* = 86) and 28% (*n* = 76), respectively.	All participants reported an improved sense of self-esteem, and for those who had experienced anxiety and depression prior to IRIS, most stated that this was reduced following the support.	The quantitative and qualitative findings indicate that IRIS support has a positive impact on mental health.	IRIS should be regarded as an effective intervention in promoting positive mental health, and its commissioning should be regarded as a justifiable return on investment.
**Genitourinary conditions**	Pre-IRIS, 27% (*n* = 72) of the sample had gynaecological problems recorded, 3% (*n* = 8) had STI recorded and 22% (*n* = 60) had UTI recorded. Post-IRIS, gynaecological problems, STI and UTI were recorded at 9% (*n* = 24) 0.4% (*n* = 1) and 8% (*n* = 22), respectively.	No participants in the qualitative phase talked of this aspect of physical health.	The quantitative results showed an improvement in recorded genitourinary conditions post-IRIS support, but this was not supported by the qualitative findings.	Qualitative studies that explore the potential impacts of IRIS on genitourinary health are recommended.
**Fatigue, pain and IBS**	Pre-IRIS, 5% (*n* = 13) had chronic fatigue recorded, 66% (*n* = 176) had pain recorded and 8% (*n* = 22) had IBS recorded. Post-IRIS, chronic fatigue, pain and IBS were recorded at 3% (*n* = 9) 20% (*n* = 53) and 2% (*n* = 6), respectively.	Some participants in the qualitative phase reported improved mobility and improvements in overall health, but not specifically in relation to fatigue, pain and IBS.	The quantitative results showed an improvement in recorded fatigue, pain and IBS post-IRIS support, but we were unable to verify this through the qualitative findings.	Qualitative studies that explore the potential impacts of IRIS on fatigue, pain and IBS health are recommended.
**Deprivation**	77% of sample cohort were from the most deprived areas of West Midlands, and 23% were living in the least deprived areas. There was a range of ethnicities within the sample cohort who had received IRIS support, with the highest number of participants (55%) identifying as white British and the next highest number (11%) identifying as British Pakistani.	We did not collect demographic data pertaining to participants’ socio-economic status, and our processes of anonymisation meant that we did not know their postcodes.	Although a broad range of ethnicities were acknowledged, the number of participants who identified as non-white were far lower than expected due to population in West Midlands containing a very cosmopolitan mix of ethnicities. More work needs to be performed to reach those communities.	DV interventions that target the most deprived communities are useful, but this must not overshadow the experiences of DV among all societal strata.
**Overall health**	The overall health was recorded as a drop post-IRIS intervention indication improvement.	Recovery is a process	The results from the quantitative phase were seen especially in mental health improving in the qualitative phase.	Intervention when provided timely and continued ongoing post-intervention has proven to show benefits to an individual’s overall health.

## Data Availability

Anonymised data are available on reasonable request via the corresponding author.
